# Development and Design of Next-Generation Head-Mounted Ambulatory Microdose Positron-Emission Tomography (AM-PET) System

**DOI:** 10.3390/s17051164

**Published:** 2017-05-19

**Authors:** Samantha Melroy, Christopher Bauer, Matthew McHugh, Garret Carden, Alexander Stolin, Stan Majewski, Julie Brefczynski-Lewis, Thorsten Wuest

**Affiliations:** 1 Industrial and Management Systems Engineering, West Virginia University, Morgantown, WV 26506, USA; smelroy@mix.wvu.edu (S.M.); gtcarden@mix.wvu.edu (G.C.); 2 Montgomery-Downs Lab, Blanchette Rockefeller Neuroscience Institute, West Virginia University, Morgantown, WV 26506, USA; cbauer5@mix.wvu.edu; 3 Mechanical and Aerospace Engineering, West Virginia University, Morgantown, WV 26506, USA; mmchugh4@mix.wvu.edu; 4 Department of Radiology, Center for Advanced Imaging, West Virginia University, Morgantown, WV 26506, USA; astolin@hsc.wvu.edu; 5 Department of Radiology & Medical Imaging, University of Virginia, Charlottesville, VA 22903, USA; sm4aa@virginia.edu; 6 Physiology & Pharmacology & Blanchette Rockefeller Neuroscience Institute, West Virginia University, Morgantown, WV 26506, USA; jblewis@hsc.wvu.edu

**Keywords:** positron emission tomography, brain, brain disorders, wearable, microdose, brain injuries, stroke, dementias, autism

## Abstract

Several applications exist for a whole brain positron-emission tomography (PET) brain imager designed as a portable unit that can be worn on a patient’s head. Enabled by improvements in detector technology, a lightweight, high performance device would allow PET brain imaging in different environments and during behavioral tasks. Such a wearable system that allows the subjects to move their heads and walk—the Ambulatory Microdose PET (AM-PET)—is currently under development. This imager will be helpful for testing subjects performing selected activities such as gestures, virtual reality activities and walking. The need for this type of lightweight mobile device has led to the construction of a proof of concept portable head-worn unit that uses twelve silicon photomultiplier (SiPM) PET module sensors built into a small ring which fits around the head. This paper is focused on the engineering design of mechanical support aspects of the AM-PET project, both of the current device as well as of the coming next-generation devices. The goal of this work is to optimize design of the scanner and its mechanics to improve comfort for the subject by reducing the effect of weight, and to enable diversification of its applications amongst different research activities.

## 1. Introduction

Positron-Emission Tomography (PET) is an imaging modality which takes advantage of radiation emitted from labeled ligands to produce a medical image of the distribution of the imaging agent in the animal or in human body. Imaging with PET is found useful in the detection of various disease states in different organs of the body, such as cancer, as well as having other applications in brain and central nervous system conditions. However, in conventional PET systems, the individual undergoing the scan must remain very still for long durations in the supine position to get useful images for analysis. This limitation, along with radiation exposure inherent in PET imaging, reduces the utility of this particular modality both clinically and in research. A more mobile, low dose variant of a PET system, that does not require patients to remain still for long periods of time would benefit specifically the imaging of patients with cancer, dementias such as Alzheimer’s or Parkinson’s disease, depression, epilepsy, stroke, or any other kind of brain injury [1,2,3,4,5].

Clinically, a significant reduction in radioactive dose with no associated decrease in image quality would have a long-reaching positive impact on many populations. A lower dose would allow more frequent use such as longitudinal scans, for example, in assessing brain changes in stroke recovery, in tumor remission monitoring, and be preferred when needed to image pediatric populations.

In addition, unique benefits will be obtained from implementation of motion-tolerant systems. Certain patients who simply cannot remain still, such as young pediatric patients, those with severe autism spectrum disorder (ASD), or epilepsy, and those with a form of dementia like Parkinson’s disease could for the first time benefit from functional medical brain imaging without being sedated. If this motion-tolerance could be expanded to include imaging in the upright position, and even during limited movement, it suddenly becomes possible to image an even more diverse clinical population. Phenomena which have not yet been possible to capture, such as how the brain responds to purposeful large-scale movement, balancing tasks and vestibular processes, or walking after stroke could be possible to obtain. Furthermore, if done with a design that permits low-dose injections, brain changes and neural metabolism might even be one day longitudinally tracked to assess therapeutic effectiveness.

From a research perspective, the opportunities such a system offers are vast and could lead to novel insights in a variety of areas. Adding the possibility of utilizing upright low-dose brain PET scanning during real world tasks such as complex, social interaction or playing an instrument or combining mobile PET imaging with virtual reality would be very powerful and open up new opportunities to better understand the mechanisms of the human brain. Signature brain changes and neural processes might be uncovered in any situation that could be adequately simulated. The imaging of certain savants or those with outstanding abilities might also be investigated to help learn more about what allows these individuals to perform at such a remarkable level.

Fortunately, this discussion is not merely academic. Early preclinical studies investigated the initial concept of a wearable PET scanner on rats [6,7]. This novel imager, RatCap, permitted PET imaging of the rat brain in real time in moving animals. Following on this success, the first wearable PET brain imager designed for humans, PET-Hat, allowed human imaging during controlled head movement while seated [8,9]. However, the PET-Hat imager has not yet been tested on humans. A recently developed imager (Helmet-Chin PET) developed by Yamaya and colleagues is designed for upright human brain imaging, has outstanding brain coverage and sensitivity, and provides high-quality imaging data of brain phantoms, but at the expense of detector bulkiness and the system not being truly “wearable” [10]. 

**Figure 1:**
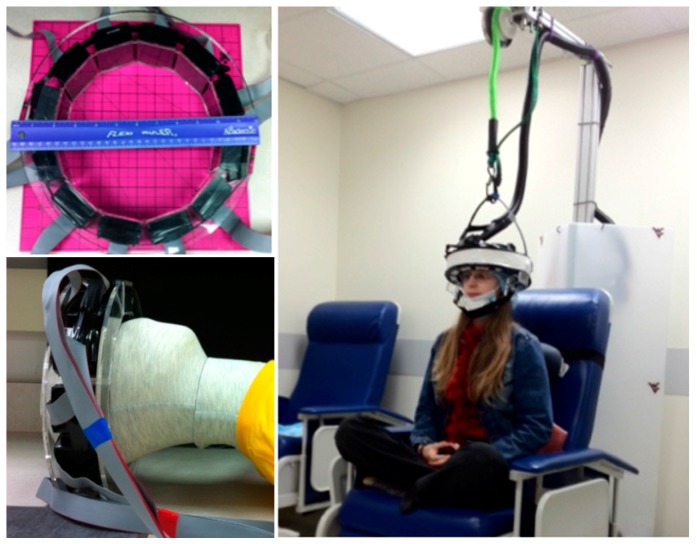
Original Helmet_PET module setup, left; AM-PET Helmet_PET in action showing green bungee cord going over pulley and support frame, right.

With these advances in imaging technology and additional advances in radiation sensors, interests have been piqued about using detector technologies to build a truly wearable device capable of producing upright brain images in humans investigating the aforementioned disorders and conditions. West Virginia University’s (WVU) ambulatory microdose positron emission tomography (AM-PET) project involves a collaboration of WVU with multiple institutions, including General Electric Global Research Center (GE GRC), Niskayuna, NY l, University of Virginia, University of Washington, Seattle, and University of California, Davis with their corresponding technical expertise. The current AM-PET small prototype is a portable, lightweight wearable brain PET scanner with a ~5 cm vertical field of view that allows a patient to complete various tasks to get pseudo-real time (~30 s intervals) images of the brain even during head motion. Furthermore, this novel system allows the use of a lower dosage of the radioactive tracers compared to full size PET scanner systems [2,11]. This device was previously shown to produce images at 25% of injected clinical dose, with an estimation that the device could image at even 10% of the standard [2]. This original AM-PET Helmet_PET imager was comprised of an acrylic support frame with attached 12 photo-detector modules positioned in the ring geometry and strapped to the head using safety helmet attachments (see Figure 1). This prototype ultimately provided the proof of concept for the ambulatory PET and delivered high-definition images of the brain as volunteers were performing simple tasks. 

One of the AM-PET project goals is to solve issues that arise with the current state of the art in brain scanning and limitations of the currently employed imaging techniques while combining motion tolerance with coverage of deep brain structures. Standard clinical PET scanners are heavy, mostly stationary, can image patients only in the horizontal position, and scans are expensive. The AM-PET concept breaks this mold with a lightweight solution that is mobile and its use is relatively inexpensive in comparison to regular PET scans [12]. 

Many challenges arise with this type of novel imager, with one of the main obstacles being the sheer weight of the 12 PET detector modules arranged on the patient’s head; the modules of the prototype weigh a total of 2.4 kg and thus an additional supporting structure to alleviate the weight of the helmet and relieve the patient’s neck/head was required. Initially the WVU team created a structure that would ease some of the weight of the helmet using a simple bungee cord design, which works well when the patient has limited head movement in a sitting or standing in place position. However, challenges can occur during more vigorous head movements in various directions. In addition, the structure of the original AM-PET Helmet_PET was such that the imager was often too loosely strapped to the head, which could result in the imager ring shifting and rotating with relation to the patient’s head. This shifting is problematic for co-registration, and when present results in skewed and blurry images. While initial scan results have demonstrated the concept of mobile brain imaging with the ability to move and complete simple tasks, i.e., tapping your foot [13], the issues with limited movement and possible fatigue from the weight of the AM-PET helmet upon the subjects’ head has been identified as possible points of intervention by the engineering team.

These challenges were brought to WVU’s Benjamin M. Statler College of Engineering and Mineral Resources (CEMR) to advance the original acrylic structure and create a Generation 1 prototype (Generation 0 being the original). The priority for this prototype was to create high definition images where the modules move with the head while also creating a support system that fully alleviates the weight of the helmet and ensures proper tight attachment to the head with appropriate registration. The purpose of this paper is to describe the design plans of a mechanical system to collect high quality images with WVU’s portable AM-PET Helmet_PET with consideration of a patient’s comfort and safety and outline the technical aspects and advantages of a generation 1 AM-PET system.

The new planned (Generation 1) prototype will be affixed to a 3D printed helmet for good fit to the head and position stability and will have a new four-tiered ring in order to allow imaging the whole brain in one helmet position. A Biodex Unweighing System (Biodex Co., Shirley, NY, USA) will be used as the mechanical support system for the PET scanning mobile helmet design, providing an unweighing system for the subjects’ comfort and safety [14]. The Biodex allows the patient to walk on a treadmill and therefore the capturing of images of brain activity during the complex task of walking is possible. The Biodex system is used regularly by researchers and medical professionals [15].

Specific goals include the design and manufacture of a system that significantly reduces the relative movement between the head and helmet (incl. the sensor module arrays) yet does not restrict the user’s head motion (more than necessary). The continuation of this work includes design review of the current model, design improvements based on the identified project requirements, and development of a working prototype. The success of this work will ultimately be measured by comparing the developed prototype to the original device and the newly developed metrics.

## 2. Materials and Methods

The methods section will discuss the original Generation 0 AM-PET Helmet_PET designed by scientists at West Virginia University (WVU) and the current planned (Generation 1) design of the AM-PET Helmet_PET. Design requirements for the systems include the following:

- The individual PET detector modules must be completely shielded from light (light-proof)
- The modules must stay at a (near) constant temperature as photodetectors used—Silicon Photomultipliers (SiPMs)—are temperature sensitive
- The modules must not move with relation to the head
- The modules’ location and orientation must be fixed in relation to one another
- The modules must be in close proximity to one another (small gaps)
- The helmet must be weight-supported on a subject for the duration of the scan (~30 min)
- The strain on the subject’s neck and head regions must be within an acceptable range based on safety standards
- The subject must be able to walk on a treadmill while wearing the AM-PET helmet.

### 2.1. Original AM-PET Helmet_PET *(*Generation 0*)*

**Figure 2:**
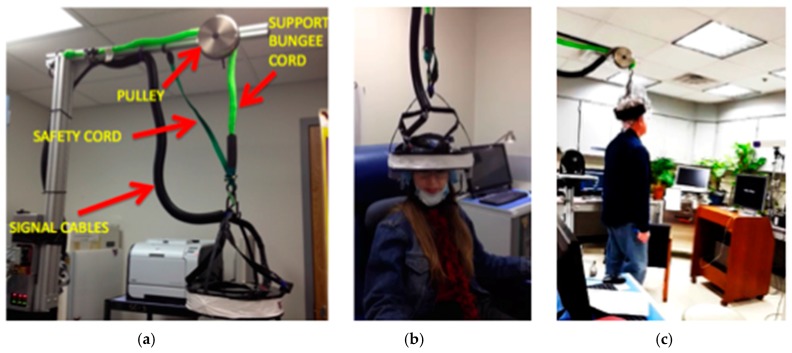
(**a**) Generation 0 support mechanics. The PET ring is attached to the support bar above, using stretchable thick cord (“bungee” type cord) going over low friction pulley (with a grove for the cord). This design accommodates limited vertical and side movements. Additional safety cord would support the weight in case flexible cord slipped off the pulley wheel; (**b**) Generation 0 can accommodate sitting subjects but also [2]; (**c**) limited situations with the patient standing, walking in place, etc.

**Figure 3:**
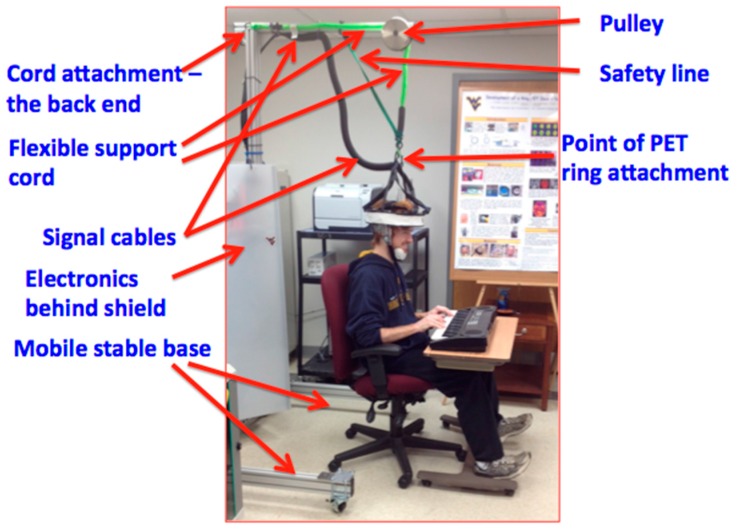
More details of the Generation 0 support mechanics.

The AM-PET Helmet_PET was developed to create a portable PET brain scanner for more individualized brain imaging as compared to the commonly known PET/CT scanner where the patient is required to remain still in an isolated environment [12,16]. The original AM-PET Helmet_PET is composed of twelve photo-detector modules evenly spaced around a patient’s head. Figure 1 displays the twelve photo-detector modules orientation with relation to the patient’s head. The modules measure 48 × 48 × 25 mm and weigh ~200 g each. The twelve modules currently weigh a total of 2.4 kg, which is too heavy for a patient to support independently over the testing period, thus a simple yet functional and safe apparatus was constructed to support this weight (see Figure 1, Figure 2 and Figure 3) [12,16].

One reason the system has low-dose imaging is due to the close proximity of the detector modules to the brain. These proximities not only alleviate the distance between the modules and the head but the distance from module to module, which should be kept to a minimum; currently the gaps are about 6–7 mm. The distance between the modules is critical because the further apart they are, the more it may degrade the resulting image.

**Figure 4:**
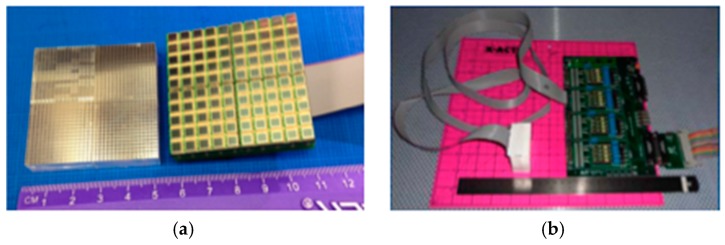
(**a**) Basic components of the original AM-PET Helmet_PET modules. Four 16 × 16 LYSO arrays were pieced together to form a 32 × 32 array. These were coupled to four 5 × 5 MPPC pixel arrays to form a 10 × 10 pixel MPPC array per module. (**b**) Electronic amplifiers were located outside of the module to reduce weight, size, power generation, and minimize magnetic field susceptibility [16].

The individual modules themselves were composed of 32 × 32 pixel arrays of 10 mm thick LYSO crystal scintillators with 1.5 mm pitch (Figure 4a; Proteus, Chagrin Falls, OH, USA) [2]. Scintillators were coupled to the compact solid-state Multi-Pixel Photon Counters (MPPCs; Hamamatsu Photonics, Hamamatsu City, Japan) via 3 mm acrylic light spreading windows. Detector readouts coupled to MPPCs for each module (AiT Instruments, Newport News, VA, USA) implemented four analog readout channels, which were ultimately relayed to the data acquisition module [17,18]. Interface modules that contained power supplies, signal circuitry and amplification stages were located outside of the modules to help reduce size and weight of the modules (Figure 4b). No active electronics except the MPPC sensors were placed in the modules. All modules were wrapped in black electrical tape to prevent light infiltration.

As mentioned previously, the helmet prototype is made of cut acrylic pieced together in a circular shape (Agile Engineering, Knoxville, TN, USA) to accommodate the twelve photo-detector modules as indicated in Figure 1. Modules are fastened around the acrylic structure and black electrical tape is wrapped over the ring structure to block light and shield the light-sensitive sensor modules. The original helmet uses a compressive fitting for the helmet with the use of an adjustable inner ring, similar to the inner mechanism of a construction safety hardhat, with an adjustable knob in the back to loosen or tighten the helmet and accommodate different head sizes. This original prototype allows a patient to sit in a chair and perform various activities such as tap a foot, or clap one’s hands, however many obstacles have occurred with the acrylic structure such as undesirable movement of the helmet with relation to the patient’s head orientation, the inability to take the modules out of the ring in a timely manner and lack of a heat shield between the patient’s head and the sensor modules (see Figure 2 and Figure 3). This has been resulting in, among other issues, increased noise present in the collected data and thus resulting images.

Due to the weight of the modules and the acrylic structure the helmet weighs around 3.6 kg in total and must have an external support system to alleviate the weight on the patient’s head to reduce the risk of injury, i.e., in the sensitive neck area. The original AM-PET Helmet_PET design uses a bungee cord attached to a sturdy frame structure to support the weight of the helmet attached to the subject’s head. The bungee cord is attached at the top-center of the helmet, to not restrict the degrees of freedom that are associated with normal head movements (i.e., rotation about the sagittal, coronal and transverse planes). However, the optimal helmet design that provides the ideal conditions to capture the most accurate readings from the PET detector modules would have virtually no relative movement between the photo-detector modules and the patient’s head. To accomplish this objective, a helmet with a tight fit on the patient’s head is crucial. If the helmet, and thus the modules, move relative to the patient’s head, motion artifacts will arise. A possible alternative option, e.g., the use of a complex real-time tracking system that adjusts the orientation automatically is not considered at this point of the development as it will add additional complexity (and weight) to the already complex system. Automated solutions will be evaluated at a later point in time, once the basic requirements are investigated and readily available solutions are implemented and tested. 

### 2.2. Generation 1 AM-PET Helmet

Building on these initial concepts, the next prototype (Generation 1) aims to advance this design and tackle the outlined challenges. Generation 1’s main objective is to improve the support system of the helmet by creating an effective support structure that frees the patient of major force due to the weight of the helmet and modules, cables, etc. A 3D printed helmet with places for the modules will use the principle of a football helmet design, with the use of an air cushion to create a better, in terms of tightness and comfort, fit for the patient and adjust for differences in head sizes and shapes. This air cushion (bladder) system on the inside of modern football helmets is designed to adjust the helmet’s fit to individual head shapes, will be used to secure the 3D printed helmet to the patient’s head. A port in the air bladder allows inflation or deflation of the bladder as needed to provide a comfortable and secure fit to the patient’s head. This gives an advantage of having a tight yet comfortable fit and thus reduced relative movement of the detector array to the patient’s head.

**Figure 5:**
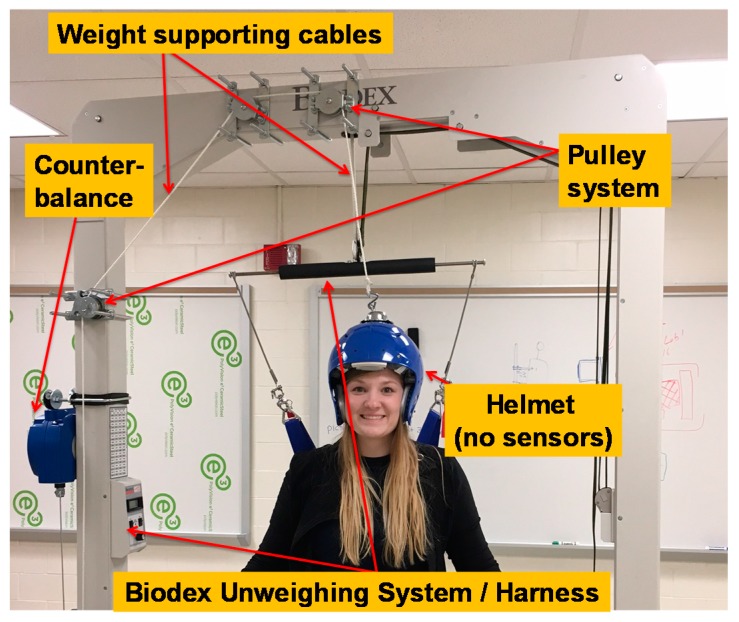
Generation 1 current support prototype with weighted helmet allowing for much more freedom. The subject/patient. Counterbalance currently supports up to 10 kg but can be upgraded. Digitizing electronics will be mounted to the support above the patient (not shown).

Generation 1’s support structure will employ a novel counterweight and pulley system (see Figure 5). This system will attach to the helmet to reduce the weight of the helmet, including the modules, cables and additional material, to create a virtually weightless experience for the patient. When the patient’s head is moving in a positive vertical direction (up), the counterweight system will retract the helmet up with a force approximately equal to the weight of the helmet. This system will also keep constant mechanical tension on the helmet so when the patient moves in the negative vertical direction (down) the helmet won’t push down on his/her head. The rotation of the head is also accounted for in this design. Generation 1 uses a ball-in-socket joint to allow the patient to rotate his/her head in all directions; and accommodate a patient’s head movement during the expected routines. This mount will attach to the center of gravity of the helmet leaving the helmet in a natural resting position allowing increased comfort of the patient and giving the patient the ability to move their head around, ideally without restriction.

## 3. Results and Discussion

**Figure 6:**
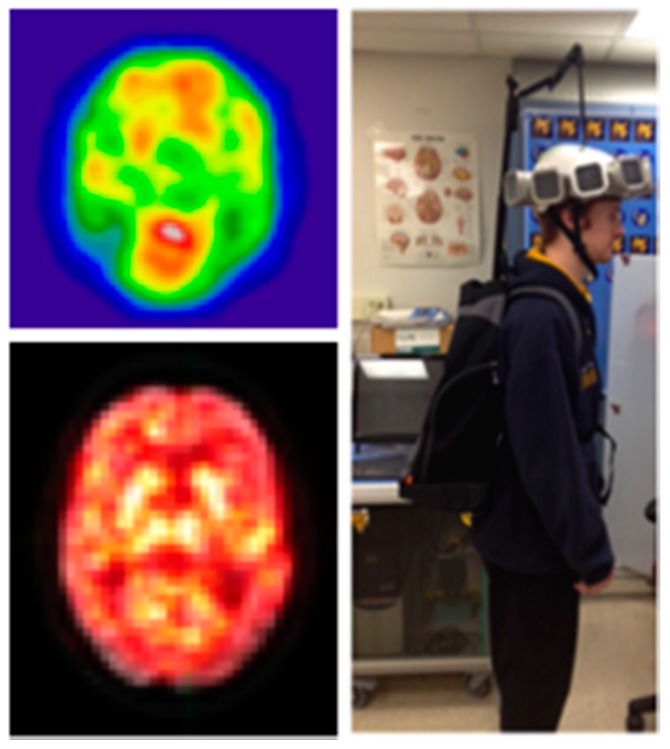
Image from the Generation 0 scanner showing a 2 mm thick brain slice after 1 min of acquisition during purposeful head rotation using ~2–3 mCi (9–12 mCi injected; imaged after 2 half-lives) of ^18^F-fluorodeoxyglucose. Elevated activity (in red) in the occipital cortex, frontal lobes, and head of the caudate can be easily identified, left top. Different 2 mm slice acquired over 10 min overlaid onto a clinical PET scan. Note the strength of signal in the head of the caudate, thalamus, and occipital region, left bottom. Original concept of the ultimate mobile imaging. Basic electronics could be stored in a backpack with backpack mounted support system taking some weight off the head, right.

In the Generation 0 imager, we were able to demonstrate that a wearable lightweight PET imager can produce the same basic pattern as clinical PET by using approximately 25% of the clinical standard dose of ^18^F^-^fluorodeoxyglucose [2]. This same basic pattern was not altered by purposeful, controlled rotation of the head by as much as ±45°. Although attachment to the head and co-registration with the brain throughout scanning was possible, it had deficiencies. However, the successful application of this early proof of concept imager lead to the AM-PET project, where ideas for next generation imagers are articulated. Figure 6 shows exemplary scanning results of the early prototype system. For more in-depth information on the results of the tests and the resultant images please consult [2].

**Figure 7:**
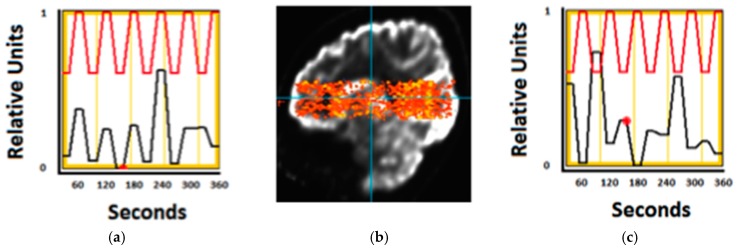
(**b**) Image from Generation 0 imager (activity in warm colors) is overlaid onto that of standard PET anatomical slice (greyscale) to show the plane of image acquisition on the head. This is a sagittal slice from front (left) to back (right) of brain, with the top of the brain at the top of the picture and cerebellum/brain stem at the bottom. Individual voxels were analyzed to see if they either increased in activity during the hand task (correlated, (**a**)) vs. foot tapping task (anti-correlated, (**c**)). Red represents an ideal model waveform, while the black represents actual intensity differences in the image due to higher count rates (averaged to one measure during each 30 s period for this analysis).

Furthermore, we have also collected preliminary PET images of the brain on human participants during purposeful movement where participants would alternate between a finger movement and tapping their foot (Six, one minute cycles of each 30 s task, Figure 7).

The imager was placed on the participant’s head while they were sitting with the scanner supported by the overhead mechanical frame and support cord. Brain activation unique to each movement in relevant brain motor network regions was seen in this pilot data set, using a voxel by voxel time series analysis. In addition, two patients performed a walking in place task, alternating walking with standing (data not shown). The patient with the scanner placed higher on the head to cover motor cortex showed leg region representation, while the second patient with lower PET placement showed basal ganglia activity, however more datasets will need to be collected to confirm the reliability of 30 s temporal resolution. This preliminary data demonstrates the proof-of-principle concept that metabolic activity can be reliably acquired from the brain during purposeful movement, which may include actual locomotion in the near future. Further improvements to mechanics and device performance, described in the upcoming paragraphs, have tremendous implications for mobile medicine, as well as new abilities to study neurological processes during complex motor tasks. Generation 1 will consist of a four-tiered ring array with approximately 18 modules in two top full rings, and 15 modules each on the lower two (partial) rings. The modules to be used in this helmet will be similar to the ones utilized in the Generation 0 system but with more regularity. 

**Figure 8:**
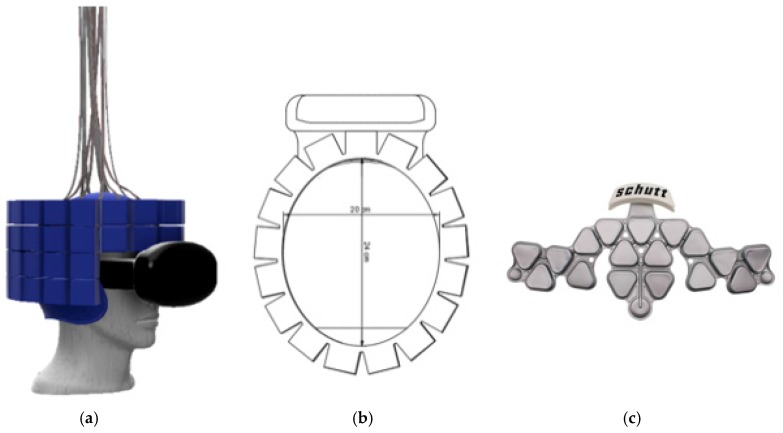
(**a**) Four-tiered 3D helmet render with VR set, 18-18-15-15 module ring; (**b**) top view of the 4-tiered ring design with dimensions; (**c**) Football helmet air bladder to be used as a concept in the 3D printed helmet for a tight, comfortable fit (Photo courtesy of Schutt Sports) [19].

Figure 8a displays this ring formation. The helmet will thus have 66 modules creating a helmet that will weigh upward of 11 kg. The helmet structure including the module arrays will be 3D printed. This manufacturing process allows for a continuous improvement method based on digital and physical mock-ups and prototype testing. A version equipped with 200 g ‘dummy’ modules (same weight and dimension as the real sensor modules) will be used to test the forces and strain caused by movements and momentum of the system on a human’s head and neck area. A measurable confirmation of the safety of the system will be made with the use of a Vicon-infrared, marker-tracking system, a Surface Electromyography (EMG) system as well as various acceleration sensors to compare the two systems in realistic scenarios. The test with the Vicon system will identify if there is any movement of the head relative to the helmet while the EMG system primarily targets possible exposure of critical stain on the patients’ neck, head or related areas by the newly designed Generation 1 helmet as the helmet is moved up and down, side to side and turned. This is done to ensure the safety of a patient at all times as well as to better understand the requirements for the final design of this system (Generation 1) but also future generations, e.g., a touchless robotic system that centers the sensor array in real-time around the patient’s head.

The AMPET family of the upright wearable brain PET imagers is enabling a new range of opportunities in studies of the human brain in the healthy and in the diseased states. They were not initially designed with performance equivalence compared to the conventional PET scanner in mind but to open completely new horizons of brain research, diagnosis and cure monitoring and guidance. Nevertheless, equivalency in basic pattern imaging has already been demonstrated to some extent, even with the initial proof-of-concept device [2], and we anticipate that improved technology will create images on par or even exceeding standard PET quality, as we are already achieving higher resolution. It is important to note that the brain is engaged differently depending if the person is upright executing behavioral tasks or placed still in the horizontal bore of the standard whole body (WB) PET scanner. In addition, the standard WB scanners were designed as holistic imagers and are not optimized to image the brain. Both the spatial resolution and sensitivity of these types of imagers are suboptimal for many brain imaging experiments. 

In order to achieve high performance and functionality expectations with tolerance to ambulatory motion, very important components include safe and functional support mechanics. This manuscript serves to communicate advances in the mechanics and implementation of this wearable brain imaging concept, which has multiple unique advantages. The limiting factor of the wearable systems is that the overall weight must be reduced which impacts the brain coverage and sensitivity of the imager. 

Table 1 illustrates the differences between a standard PET scanner system and the upright imagers (HELMET_PET system and Gen 1 & 2) developed within the AMPET project. The table furthermore presents the advances already achieved and/or expected by the new systems.

### 3.1. Module and Helmet Design

The 3D printed helmet is the main structure for the next generation ring of PET detector modules. The helmet has multiple advantages including a tight fit through the use of an air bladder to adapt and adjust to each individual’s head shape and the helmet’s sturdiness to support the weight of the larger number of modules as well as a better, more evenly distribution of weight. The modules form either an elliptical or round ring around the helmet with as close proximity as manageable to allow for images without artifacts. A swivel mount will be attached to the top of the helmet to allow for movement of the patient’s head in all directions. The mounting point is placed on the vertical line going through the helmet’s center of gravity; therefore, it does not affect the patient’s resting head movements. In our plan, we assume that the individual PET modules can be taken out of the ring easily by removing the screws around the “lid” and taking the module out of the 3D printed structure. This allows for easy maintenance of the modules and redistribution in case of unusually big/small head sizes, which are not covered by the standard helmet. Future manufacturing designs will involve a plastic mold with an easy to use “door” and a clip so there are no screws that can be dropped, and thus improved accessibility.

The current design for Generation 1 is a four-tier ring that will be able to scan the entire brain. The front of the helmet will be open to allow the patient to see and have the ability to use the VIVE Virtually Reality (VR) set; Figure 8a,b display the rings arranged around the helmet structure including the VR headset. The virtual reality set will allow more broad stimulations of the patient from going on a roller coaster to taking a hike or exposing them to certain, unusual situations and stimulations in the virtual space. Along with the helmet’s detector modules arranged in the described rings, come several wires which required to connect each sensor module to the analyzing readout. This creates a complex array of cables emerging from all over the surface of the helmet. The approximate combined number of cables to be managed for one generation 1 system is estimated to be over 450 cables total. Thus, effective and efficient cable management is a critical requirement of the design and development process that needs to be tested virtually, in the digital mock-up and especially once a physical 3D helmet is created, adding weight and a non-trivial additional design challenge.

The helmet will be modular and able to fit the majority of people’s head shapes and sizes. Using the 99th percentile head sizes as a reference, the helmet has been designed to fit someone’s head from 16.6 cm in width (side to side) and 21.7 cm in thickness (from front to back) [20]. The helmet has room for an air bladder to fit someone with this size of head and through interchangeable air bladders the helmet will also be able to fit someone with a much smaller head, yet keep the stability of the helmets integrity, see Figure 8c. The air bladder is an integral part of the helmet’s safety; it is essential that the helmet does not shift relative to the head. If shift does transpire it could cause multiple problems including whiplash and resulting in serious injury of the patient. The smaller the patient’s head, the larger the air bladder must be to fill in the head to helmet gap. In case this is done improperly, more shift could occur and thus more acceleration of the helmet and modules with relation to the head. This leads to the modular design and thus the option of having additional helmet structures available for unusually large or small heads, that would lead to too much of a gap to be filled by the air bladder.

The planned number and distribution of modules is sufficient to cover 99th percent of head sizes with relatively no gaps between the modules. Each tier of the ring is the same size and shape in an elliptical perimeter. The elliptical perimeter follows the shape of the head ideally and can easily be manipulated to add the necessary modules in a preferable pattern. The 18 modules in the top two rings allow for minimal gaps (ideally zero) and the bottom two follow the same pattern although the front three modules are eliminated to allow a patient’s vision forward and down to be unobstructed. The elliptical shape of the ring will be approximately 24 cm in length and 20 cm in width. This allows for a 2 cm air bladder to transpire between someone with a larger head and allow for the modules to have relatively no gaps between them. The helmet will be supported by the modified Biodex system. The helmet will be “hooked” through the swivel mount to a rope that will transpire directly above the patient’s head. This will eliminate force in the up and down motion.

### 3.2. Biodex Unweighing System

**Figure 9:**
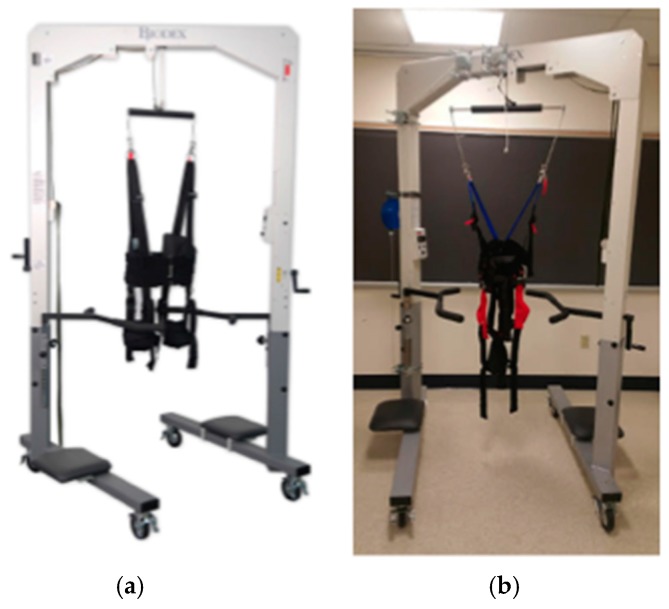
(**a**) Unmodified Biodex system (Photo courtesy of Biodex Medical Systems, Inc. [14]); (**b**) modified Biodex system with extended bar attached to the harness, the counterbalance to the left and the four pulleys.

**Figure 10:**
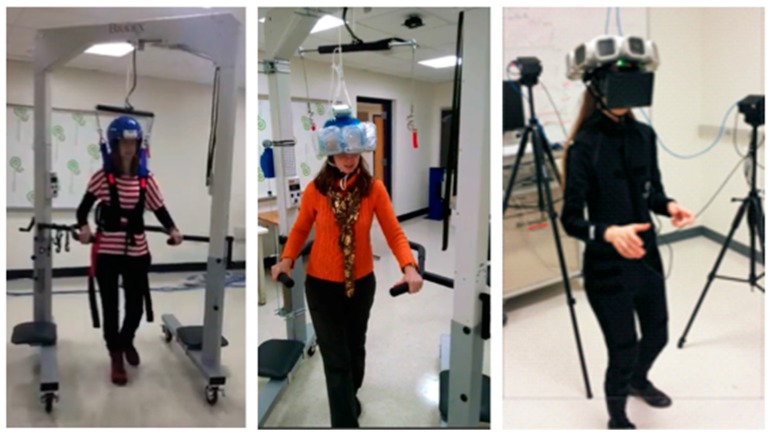
The Generation 1 fully mobile upright safe design using counterbalanced weight support of the adapted Biodex system allows for the subject can push the wheeled support to move along. In the center is shown the (passed) realistic test with a 10 kg mockup helmet (with lead weights used instead of detector modules simulation similar volume and weight compared to the functional sensor modules incl. casing and cables). The subject could also sit or stand, or even walk with VR goggles.

The Biodex unweighing system was designed for physical therapy treatments to help those who have difficulties in walking (or need to build up strength), to give weight support as a patient walks on a treadmill or around a room. Individuals fit inside a harness that is attached to the Biodex, with the Biodex as the structural support so that the patient can walk with part or all of their weight supported (adjustable). The AM-PET team is using the harness provided by the Biodex for the safety of the patient and modules, in case of a fall; this system can support weight up to 136 kg giving a large range of people the ability to use the system. The helmet itself will weight upward of 10 kg and will use the Biodex system as the structural support of the helmet as well as safety for the patient in the case of a fall, see Figure 9 and Figure 10. The design of the Biodex system was used to meet the goal of allowing the patient wearing the helmet to use all degrees of freedom and to have the helmet feel nearly weightless. Another advantage of the use of the Biodex is that it allows for nearly unrestricted walking on a treadmill, but also has wheels on the bottom to allow the patient to walk around for future designs. The design meets the needs of Generation 1 system in many different ways, per our modification. The counterweight system that we added performed as intended to move with the helmet up and down while taking the weight of the helmet off the patient. Instead of the use of a common counterweight, as typically used in exercise equipment, the new AM-PET design uses a counterbalance where the weight setting can be manually changed to the weight of the helmet, internally. The counterbalance was placed on the side of the Biodex system with its cable threaded through a system of pulleys. The pulleys are used to guide the cable towards the center of the helmet. The team made minor modifications to the Biodex system. The system has a bar above the harness, which serves the purpose of a single point suspension and permits functional pelvic rotation and versatility when walking. The original bar’s length was too short compared to the diameter of the helmet, and thus was lengthened. The attachment rings are now further outside of the helmet allowing for the correct functional use. The helmet will attach to a hanging support cable; this cable can be easily lowered by pulling an attached helmet down to accommodate multiple heights, on and off a treadmill and while in a sitting position. Figure 9a is an image of the unmodified unweighing system, and Figure 9b is the modified Biodex system with the prototype counterbalance system installed to support the helmet with the detector array. Figure 10 depicts experiments with the prototype mechanical support and mockup AMPET Helmet_PET system as well as a person emerged in a virtual reality environment that can be utilized for AMPET studies.

With the most current design of the helmet, weight management was certainly a key consideration in creating the support system of the helmet. To reduce any undesirable stress on the neck of the patient, the supporting system utilizes a counterbalance. In the image below there is a blue box to the left of the Biodex with the wire cable acting downward and managed through a set of pulleys; this allows for an internal system that acts as a counterweight. This counterbalance is to offset the weight of the helmet that could be felt on the patient’s neck. Counterweights can be found in all sorts of mechanical devices to make lifting a load more efficient. An example can be found in elevators. In this instance, the counterweight would balance out the load of the elevator so the motor did not have to lift the entire elevator. Depending on the location of a counterweight, it will provide the necessary action to reduce the overall weight of what needs to be lifted. In this supporting system, the counterweight is running through a series of pulleys and attaches to the top of the helmet. This positioning will act as if the counterweight was directly above the helmet. When set at the same weight as the helmet, a successful counterweight system will balance out the weight so there is little to no pressure on the patient. It is important to set it to the weight of the helmet to eliminate the system to pull or push down on the patient causing any unwanted pressures. In this application, if the patient’s head travels in an upward motion, the counterweight will retract the cable to lift the helmet. When the patient’s head is traveling in a downward motion, the release just enough cable to not restrict the patient’s movements and still hold the helmet so it is not pressing down on the head and neck area.

This counterbalance will allow for patients to have weight of the helmet alleviated while walking on a treadmill, walking around a room and sitting in a chair. The average person walks with most movement in the upwards and downwards motion (with little side to side motion) [21] giving this counterbalance a simple, yet practical, weight reducer.

Future designs will consider the specific requirements of people with Parkinson’s disorder, as there are a few different irregularities that can occur as they walk. First, the walking pace can slow down as they shuffle their feet and a common forward leaning position is assumed. Freezing can also occur, which includes the sensation of not being able to move their feet, like they are frozen to the ground. The above issues compound to bring people suffering from the disease a higher risk of falling [22]. This type of gait needs to be included in the design to ensure that the patients are not subject to injury from resulting falls or unusal movements, while being equipped with the AMPET helmet and strapped in the Biodex Unweighing system.

### 3.3. Future for AM-PET

The current system has several limitations, such as the inability to walk through a normal doorway (due to the size of the Biodex frame), non-uniform support when patients head is tilted (due to the upwards force only), and the heat sensitivity of the modules themselves (creating non-uniformities in the SiPM modules) (see Table 1 for overview). While the original design allowed for a patient to sit in a chair, the current design would allow a patient to walk on a treadmill or slowly forward, with the vision of future designs being completely portable. Even the design here would be very useful for research of social interactions and behaviors in a virtual reality environment, and a more portable version would extend these applications. Some options for a completely portable design include use of a robotic arm with sensors, such as accelerometers, to move along with the head for a virtually weightless system.

Another area in which the Generation 1 design aims to improve upon is keeping the sensor modules at a constant temperature during a measurement as the changing temperature inside the module, especially rising temperatures, results in image distortion. As of today, three potential sources for sensor modules temperature increase are identified:

1. The patient’s head (body temperature) (*external*)
2. Scanning room (environment) temperature (*external*)
3. Sensor electronics heat dissipation (*internal*).

Therefore, the temperature control is not trivial, especially as the target temperature change during a measurement cycle should not exceed 0.5 degrees Celsius, ideally even lower. This might require an active temperature control system within each individual module casing.

Overall, AM-PET is a revolutionary brain imaging tool to allow neuro-researchers to study the neuro-correlations of active behavior, and discover new mechanisms and targets for brain’s normal operation and during disorders and how other aspects of brain research is conducted. We believe that AM-PET helmet will have impact on medical care with the ability for early detection of cancer, dementia and other diseases in the brain.

## Figures and Tables

**Table 1:** Comparison between standard PET scanner and upright imagers.

Imager	General Feature	Geometry	Motion Friendly	Spatial Resolution	Sensitivity %	Axial Coverage	Support Mechanics
WB PET scanner	Standard of Care	Horizontal Supine or Prone	No	4–5 mm	1	Whole brain	Bed
Helmet_PET	Wearable Feasibility Prototype	Upright	Yes	2–4 mm	0.5	~5 cm	Passive Vertical Weight Support
AMPET First Generation	Dissemination Prototype	Upright	Yes	2–5 mm	3–5	Whole brain	Passive Weight Support
AMPET Next Generation	Dissemination Prototype	Upright	Yes	2–3 mm	5–10	Whole brain	Active Weight Support
